# Supplementary material for: Drug repurposing for aging research using model organisms
Source: Aging Cell. 2017 Jun 16;16(5):1006–15. doi: 10.1111/acel.12626 (PMC5595691; doi:10.1111/acel.12626)
Supplement: Supplementary file 7 — Data S1 Zip‐Archive of all report cards. [file ACEL-16-1006-s007.zip › RC_ASW.pdf]

ASW

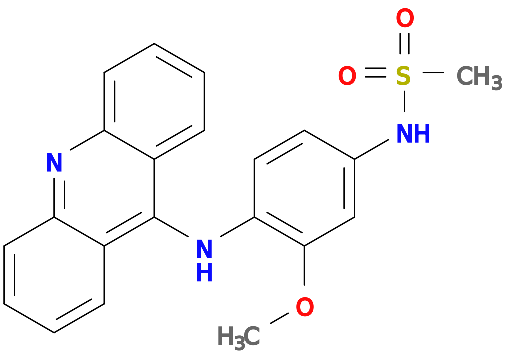

Database identifiers

ChEMBLCompound	CHEMBL43
DrugBank	DB00276
CHEBI	2687
eMolecules	35870385

Ranking

	Rank	Score
Drosophila	447/697	0.321
C. elegans	329/591	0.181

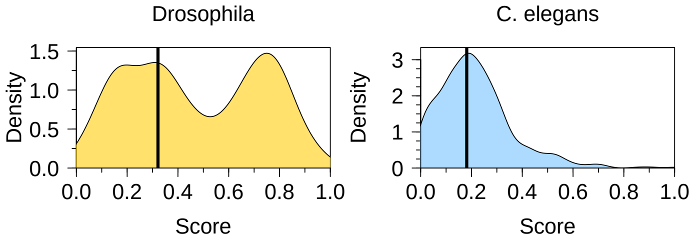

	Ageing implication	Domain conservation	Binding site conservation	Binding affinity	Bioavailability	Lipinski	Promiscuity	Purchasability	Drug approval	**Total**
Drosophila	0.624	0.963	0.851	0.428	(0.9)	-0.05	-0.0	0.1	0.075	0.321
C. elegans	0.624	0.963	1.0	0.428	0.221	-0.05	-0.0	0.1	0.075	0.181

Names

- **amsacrine**
- 4'-(9-Acridinylamino)-3'-methoxymethanesulfonanilide
- 4'-(9-Acridinylamino)methanesulfon-m-anisidide
- 4'-(9-Acridinylamino)methanesulfon-meta-anisidide
- Amsidyl
- M-amsa

Roles

antineoplastic agent, EC 5.99.1.3 [DNA topoisomerase (ATP-hydrolysing)] inhibitor

Status

Approved drug (according to ChEMBL)	No
Classification (according to DrugBank)	approved
Administration Route	Intravenous
Number of Rule of 5 violations	1
Binding affinity to original target in log units (RF-Score prediction)	4.71
Burns *C. elegans* bioavailability prediction	-4.08

Medical Information

Indicatation: For treatment of acute myeloid leukaemia.
Mechanism of action: Amsacrine binds to DNA through intercalation and external binding. It has a base specificity for A-T pairs. Rapidly dividing cells are two to four times more sensitive to amsacrine than are resting cells. Amsacrine appears to cleave DNA by inducing double stranded breaks. Amsacrine also targets and inhibits topoisomerase II. Cytotoxicity is greatest during the S phase of the cell cycle when topoisomerase levels are at a maximum.
Toxicity: Symptoms of overdose include nausea and vomiting, diarrhea, some cardiotoxicity (rarely).
Metabolism: Extensive, primarily hepatic, converted to glutathione conjugate.

(Information from DrugBank)

Compound Target Characteristics

DNA topoisomerase 2-beta

Best gene implication in ageing for this target family came from gene F1M7A0 via mapping the annotation from RGD 62048 annotated in RGD 2014-03-11. Annotation GO 7568 (aging) was Inferred from Expression Pattern

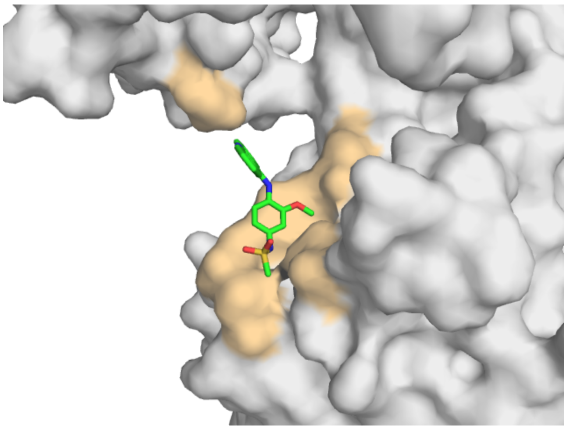

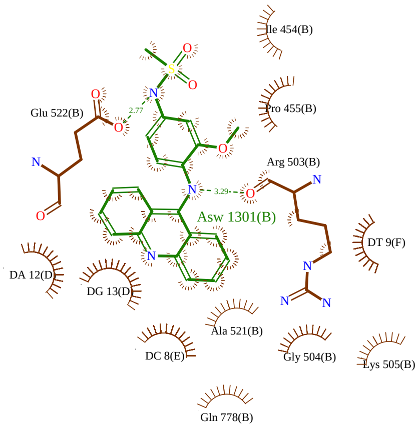

protein	amino acids contacts (binding site)
PDB:4g0u:chainA:Q02880	P R G K A E Q
tr:E9PCY5:E9PCY5_HUMAN	P R G K A E Q
sp:Q02880:TOP2B_HUMAN	P R G K A E Q
tr:F1M7A0:F1M7A0_RAT	P R G K A E M
sp:Q64511:TOP2B_MOUSE	P R G K A E Q
tr:Q6NR40:Q6NR40_DROME	P R G K A E V
sp:P15348:TOP2_DROME	P R G K A E V
sp:Q23670:TOP2_CAEEL	P R G K A E Q
tr:E9P8H0:E9P8H0_YEASX	P R G K A E Q
tr:E9P8H2:E9P8H2_YEASX	P R G K A E Q
tr:E9P8H4:E9P8H4_YEASX	P R G K A E Q
tr:E9P8H5:E9P8H5_YEASX	P R G K A E Q
tr:E9P8H7:E9P8H7_YEASX	P R G K A E Q
tr:E9P8H8:E9P8H8_YEASX	P R G K A E Q
tr:Q7LMA0:Q7LMA0_YEASX	P R G K A E Q
tr:E9P8G9:E9P8G9_YEASX	P R G K A E Q
sp:P06786:TOP2_YEAST	P R G K A E Q

	whole protein		domain-based		contact-based	
protein	ident	simil	ident	simil	ident	simil
PDB:4g0u:chainA:Q02880	1.0	1.0	1.0	1.0	1.0	1.0
tr:E9PCY5:E9PCY5_HUMAN	0.71	0.71	1.0	1.0	1.0	1.0
sp:Q02880:TOP2B_HUMAN	1.0	1.0	1.0	1.0	1.0	1.0
tr:F1M7A0:F1M7A0_RAT	0.64	0.83	0.87	0.96	0.86	0.84
sp:Q64511:TOP2B_MOUSE	0.96	0.98	0.99	1.0	1.0	1.0
tr:Q6NR40:Q6NR40_DROME	0.41	0.58	0.75	0.93	0.86	0.85
sp:P15348:TOP2_DROME	0.47	0.71	0.75	0.93	0.86	0.85
sp:Q23670:TOP2_CAEEL	0.45	0.73	0.73	0.92	1.0	1.0
tr:E9P8H0:E9P8H0_YEASX	0.34	0.64	0.58	0.86	1.0	1.0
tr:E9P8H2:E9P8H2_YEASX	0.34	0.64	0.58	0.86	1.0	1.0
tr:E9P8H4:E9P8H4_YEASX	0.34	0.64	0.58	0.86	1.0	1.0
tr:E9P8H5:E9P8H5_YEASX	0.34	0.64	0.58	0.86	1.0	1.0
tr:E9P8H7:E9P8H7_YEASX	0.34	0.64	0.58	0.86	1.0	1.0
tr:E9P8H8:E9P8H8_YEASX	0.34	0.64	0.58	0.86	1.0	1.0
tr:Q7LMA0:Q7LMA0_YEASX	0.34	0.64	0.58	0.86	1.0	1.0
tr:E9P8G9:E9P8G9_YEASX	0.34	0.64	0.58	0.86	1.0	1.0
sp:P06786:TOP2_YEAST	0.34	0.64	0.58	0.86	1.0	1.0

Top2 (FBgn0003732) associated phenotypes

developmental rate defective, heat sensitive, lethal - all die before end of first instar larval stage, lethal - all die before end of second instar larval stage, lethal - all die during first instar larval stage, male fertile, neuroanatomy defective, partially lethal - majority die, small body, somatic clone

(Information from FlyBase)

Top2 (UniProt:Q6NR40) annotation

Function: Control of topological states of DNA by transient breakage and subsequent rejoining of DNA strands. Topoisomerase II makes double-strand breaks. (RuleBase:RU000381).
Subunit: Homodimer. ECO:0000256—RuleBase:RU000381.

(Information from UniProt)

Top2 (UniProt:P15348) annotation

Function: Control of topological states of DNA by transient breakage and subsequent rejoining of DNA strands. Topoisomerase II makes double-strand breaks.
Cofactor: Mg(2+) Evidence=(PROSITE-ProRule:PRU00995); Name=Mn(2+); Xref=ChEBI:CHEBI:29035; Evidence=(PROSITE-ProRule:PRU00995); Name=Ca(2+); Xref=ChEBI:CHEBI:29108; Evidence=(PROSITE-ProRule:PRU00995); Note=Binds two Mg(2+) per subunit. The magnesium ions form salt bridges with both the protein and the DNA. Can also accept other divalent metal cations, such as Mn(2+)

or Ca(2+). (PROSITE-ProRule:PRU00995);
Subunit: Homodimer.
Subcellular location: Nucleus.
Miscellaneous: Eukaryotic topoisomerase I and II can relax both negative and positive supercoils, whereas prokaryotic enzymes relax only negative supercoils.

(Information from UniProt)

K12D12.1 (UniProt:Q23670) annotation
Function: Control of topological states of DNA by transient breakage and subsequent rejoining of DNA strands. Topoisomerase II makes double-strand breaks (By similarity).
Cofactor: Mg(2+) Evidence=(PROSITE-ProRule:PRU00995); Name=Mn(2+); Xref=ChEBI:CHEBI:29035; Evidence=(PROSITE-ProRule:PRU00995); Name=Ca(2+); Xref=ChEBI:CHEBI:29108; Evidence=(PROSITE-ProRule:PRU00995); Note=Binds two Mg(2+) per subunit. The magnesium ions form salt bridges with both the protein and the DNA. Can also accept other divalent metal cations, such as Mn(2+) or Ca(2+). (PROSITE-ProRule:PRU00995);
Subunit: Homodimer.
Subcellular location: Nucleus
Miscellaneous: Eukaryotic topoisomerase I and II can relax both negative and positive supercoils, whereas prokaryotic enzymes relax only negative supercoils.

(Information from UniProt)
